# TGF-***β*** Superfamily Receptors—Targets for Antiangiogenic Therapy?

**DOI:** 10.1155/2010/317068

**Published:** 2010-05-13

**Authors:** Jasmin Otten, Carsten Bokemeyer, Walter Fiedler

**Affiliations:** Sections of Pneumonology and Bone Marrow Transplantation, Department of Oncology and Hematology, Hubertus Wald University Cancer Center, University Medical Center Hamburg-Eppendorf, 20246 Hamburg, Germany

## Abstract

The TGF-*β* pathway controls a broad range of cellular behavior including cell proliferation, differentiation, and apoptosis of various cell types including tumor cells, endothelial cells, immune cells, and fibroblasts. Besides TGF-*β*'s direct effects on tumor growth and its involvement in neoangiogenesis have received recent attention. Germline mutations in TGF-*β* receptors or coreceptors causing Hereditary Hemorrhagic Teleangiectasia and the Loeys-Dietz syndrome underline the involvement of TGF-*β* in vessel formation and maturation. Several therapeutic approaches are evaluated at present targeting the TGF-*β* pathway including utilization of antisense oligonucleotides against TGF-*β* itself or antibodies or small molecule inhibitors of TGF-*β* receptors. Some of these therapeutic agents have already entered the clinical arena including an antibody against the endothelium specific TGF-*β* class I receptor ALK-1 targeting tumor vasculature. In conclusion, therapeutic manipulation of the TGF-*β* pathway opens great opportunities in future cancer therapy.

## 1. TGF-***β*** Pathway

The TGF-*β* superfamily consists of over 30 structurally related multifunctional proteins, including three TGF-*β* isoforms (TGF-*β*1, 2, and 3), three forms of activin, and over 20 bone morphogenic proteins (BMPs), which control a broad range of cellular behavior such as cell growth, differentiation and apoptosis in various cell types including tumor, immune, and endothelial cells as well as fibroblasts [1–5].

 Ligand signaling is mediated through two related single transmembrane type I and type II receptors, which together comprise the only known family of serine/threonine kinases [6–8]. In mammals, there are five different type II (TGFBR2, ActR-IIa, ActR-IIb, BMPR2, AMHRII) and seven type I receptors, also named activin receptor-like kinases (ALK-1-7) [7, 9]. In most cases, the receptor combination is important for the binding of a specific ligand, but the TGF-*β* family members often bind to more than one type II and type I receptor combination [10]. Upon ligand binding, the type I and type II receptors form a heteromeric complex, presumably consisting of two type I and two type II receptors. The type II receptor exhibits a constitutively active kinase which transphosphorylates and activates the type I receptor in a glycine- and serine-rich region known as GS-box [11]. The activated type I receptor propagates the downstream signaling by phosphorylating specific receptor-regulated SMAD proteins (R-SMAD) [12, 13]. R-SMADs interact with SMAD-4, the only known common mediator SMAD (CoSMAD) in mammals, and form heteromeric complexes which translocate to the nucleus where they influence gene expression (by binding to the DNA and acting as transcription factors, coactivators, and corepressors) [14–17].

 The TGF-*β* pathway has several feedback mechanisms, which regulate the duration of the signaling. One of the feedback mechanisms is mediated by inhibitory SMADs (I-SMAD), in humans SMAD-6 and SMAD-7, which compete with the R-SMADs for binding to the type I receptor, but without the ability to transduce the downstream signal. I-SMADs also recruit the E3 ubiquitin ligases SMAD ubiquitin related factor-1 and -2 (Smurf-1 and -2), which ubiquitinate the SMADs and type I receptors, resulting in protein degradation [18–23].

**Figure 1:**
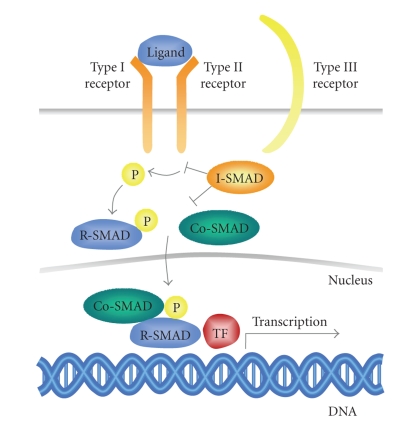
TGF-*β* signaling cascade. Upon ligand binding the constitutively active kinase of the type II receptor transphosphorylates and activates the type I receptor. Type III receptors lack any kinase activity but they act as accessory receptors and facilitate ligand binding to the type I and II receptors. Downstream signaling is mediated via R-SMADs which are phosphorylated by the activated type I receptor and form a complex with CoSMADs. This complex translocates to the nucleus where it induces transcription of downstream signaling. I-SMAD proteins represent important negative feedback structures, since they can block the signaling via competitive binding to the type I receptors or R-SMADs. R-SMAD: receptor-regulated SMAD; CoSMAD: common mediator SMAD; I-SMAD: inhibitory SMAD; TF: transcription factor.

 In humans, two accessory TGF-*β* superfamily receptors have been described which have a more indirect role in TGF-*β* signaling: betaglycan and endoglin. The later is mainly expressed in endothelial cells [24–26]. These type III receptors are structurally related transmembrane receptors with short intracellular domains that lack any enzymatic motif but contain many serine and threonine residues. They facilitate the binding of ligand to the type I and type II receptors [27]. A soluble form of endoglin has been described, most likely generated by proteolytic shedding, that antagonizes the membrane bound form [28]. The components of the TGF-*β* pathway are shown schematically in Figure 1.

## 2. TGF-***β*** Signaling in Cancer

### 2.1. Hereditary Cancer Syndromes

Several hereditary cancer syndromes with mutations in TGF-*β* superfamily members are known. The autosomal dominant familial juvenile polyposis syndrome (JPS) is the most common of the hamartomatous syndromes which occurs with an incidence of about one per 100.000 births [29]. Patients develop numerous polyps not only in the colon or rectum but also in the proximal gastrointestinal tract. Although most juvenile polyps are benign, malignant transformation occurs with a lifetime risk of colorectal carcinoma of approximately 70%. In addition, the risk of pancreatic, gastric, and duodenal carcinoma is increased [29]. Germline mutations in different members of the TGF-*β* superfamily have been described in JPS. In every fourth patient a mutation in the type I receptor ALK-3 (BMPR1A) is found [30]. In 15% of cases SMAD-4 is mutated [30]. Furthermore, mutations in the endoglin gene have been described, but the incidence is unknown [31]. 

 Hereditary nonpolyposis colorectal cancer (HNPCC) is the most common hereditary predisposition for the development of colorectal cancer. HNPCC results from germline mutations within genes involved in the DNA mismatch repair system, leading to microsatellite instability. Since the TGFBR2 gene contains a 10-base pair polyadenine repeat microsatellite sequence, it is an apparent target for inactivation caused by errors of the DNA mismatch repair. Indeed, a mutated form of TGFBR2 can be observed in up to 80% of colon cancer patients with HNPCC [32, 33].

 The autosomal cancer syndrome Cowden Syndrome (CS) and Bannayan-Riley-Ruvalcaba (BRR) disease are normally associated with a phosphatase and tensin homolog (PTEN) gene mutation. However, in one patient with CS and BRR symptoms but without PTEN mutation an ALK-3 mutation was found [34].

### 2.2. Dysregulated Expression in Cancer Patients

For several pathologies, especially cancer, a correlation between the expression level of a TGF-*β* superfamily member and the severity of the related disease has been identified, which makes the concerning TGF-*β* family member a diagnostic, prognostic, or predictive marker. 

#### 2.2.1. Transforming Growth Factors

In 1986, Nishimura et al. detected elevated TGF-*β* levels in the urine of patients suffering from advanced cancer stages compared to healthy donors [35]. Since then, increased serum levels of TGF-*β*1 have been implicated as a prognostic marker of advanced disease and poor prognosis in multiple cancer types such as gastric carcinoma, colorectal cancer, bladder carcinoma, prostate cancer, breast cancer, lung cancer, esophageal adenocarcinoma, and melanoma [36–44]. But nevertheless TGF-*β* levels are not yet used as tumor markers in clinical routine.

#### 2.2.2. Bone Morphogenic Proteins

Bone morphogenic proteins can also serve as prognostic markers, since the BMP-7 expression is increased in malignant melanomas and their metastases, which correlates with a shorter time to tumor recurrence [45]. Furthermore, high BMP-6 levels predicted development of distant metastasis in primary prostate cancer [46]. On the other hand, the mRNA level of BMP-2 was significantly decreased in breast cancer tumors compared to normal breast tissue [47].

#### 2.2.3. TGF-*β* Receptors

The expression of TGF-*β* superfamily receptors within tumor cells can be a prognostic marker. Reduced ALK-5 and TGFBR2 expression correlates with a shorter survival rate of colon cancer patients, as does reduced expression of the coreceptor betaglycan in breast and prostate cancer patients [48–50]. Low expression levels of TGFBR2 have been observed in patients with chronic myeloid leukemia [51]. In addition, mutations in ALK-5 and TGFBR2 have been described for other haematological malignancies, but it seems to be a rare event [52, 53]. A significant association between loss of BMPR2 expression and tumor grade was found in bladder transitional cell carcinoma [54]. In contrast, high expression of type III coreceptor endoglin was mainly detected on immature blood vessels in prostate tumors and had a negative impact on patient's survival as well as with response rates in breast cancer or cervical cancer [55–58]. Calabro et al. detected elevated levels of soluble endoglin that correlated with low TGF-*β*1 levels in patients with acute myeloid leukemia or chronic myeloproliferative disorders [59]. However none of these markers is used in routine clinical practice.

## 3. TGF-***β*** Signaling in Endothelial Cells

Several members of the TGF-*β* superfamily are expressed in endothelial cells and play an important role in angiogenesis and vasculogenesis. The targeted inactivation of TGF-*β* signaling components in mice revealed the pathway's crucial role in vascular morphogenesis. For example, animals lacking TGF-*β*1, ALK-5, ALK-1, endoglin, or various SMAD proteins die at midgestation during embryogenesis due to defects in vascular development of the yolk sac [10, 60–62].

 In humans, the Hereditary Hemorrhagic Teleangiectasia (HHT, also named Rendu-Osler-Weber syndrome) is an autosomal dominant disease in which vascular dysplasia results in teleangiectasia and arteriovenous malformations. Two forms with different clinical characteristics have been described: HHT type 1 patients have a mutation in the endoglin gene whereas HHT type 2 is characterized by a mutation in the ALK-1 gene. Together these mutations account for about 80% of all HHT patients [6, 63–65]. In 2005, another autosomal dominant syndrome with mutations in TGF-*β* receptors was described: the Loeys-Dietz syndrome. Patients have a very high risk for aortic dissection or rupture. Analysis of 52 families with a history of Loeys-Dietz syndrome revealed somatic mutations either in the type I receptor ALK-5 or in the type II receptor TGFBR2 [66, 67]. 

### 3.1. Functional Aspects of TGF-*β* Signaling in Endothelial Cells

In endothelial cells the type I TGF-*β* receptors, which have been investigated most thoroughly, are ubiquitously expressed ALK-5 and endothel-specific ALK-1. Previously, it was believed that ALK-5 and ALK-1 had opposite roles in angiogenesis and might balance the activation state of endothelium. Several investigators observed increased proliferation and migration when the TGF-*β*/ALK-1 pathway had been stimulated whereas stimulation of the TGF-*β*/ALK-5 pathway led to inhibition of endothelial cell proliferation and migration [68, 69]. This opposing effect was thought to be mediated by activation of SMAD-1/5/8 by ALK-1 and SMAD-2/3 by ALK-5 [68]. Due to activation of different intracellular pathways, specific changes in gene transcription can be observed. Goumans et al. revealed that the inhibitor of DNA binding 1 (ID-1), a helix-loop-helix (HLH) protein that can form heterodimers with members of the *basic HLH* family of *transcription factors*, is a specific downstream signal of ALK-1, whereas the proteinase inhibitor plasminogen activator inhibitor-1 (PAI-1) is induced by ALK-5 activation [68].

 More recently published data might alter the presumed relationship between ALK-1 and ALK-5. David et al. showed that not TGF-*β*1 but the bone morphogenic proteins 9 and 10 are likely to be the physiological ligands for ALK-1. Binding of BMP-9 to the ALK-1 and BMPR2 complex potently inhibited endothelial cell proliferation and migration [70]. The increase of angiogenesis in ECs upon ALK-1 activation in former studies was due to TGF-*β*1 binding to the ALK-1/TGFBR2 complex. Thus, the role of ALK-1 is dependant of type II receptor expression and ligand availability. Interestingly, both pathways signal via activation of SMAD-1, -5, and -8 although these SMADs have been described as characteristic BMP downstream signals [71]. Hence, additional elements must be involved in regulation of the ALK-1 pathway driving it either to the pro or antiangiogenic direction. Indeed, cross-talk between the TGF-*β* pathway with other pathways such as the mitogen-activated protein kinase (MAPK), the phosphatidylinositol-3 kinase (PI3K) or the Hedgehog pathways have been described [72].

 Very recently, a possible explanation for the requisite role of ALK-1 and ALK-5 in angiogenesis has been described. Shao et al. demonstrated that ALK-1 and ALK-5 are both essential for the regulation of vascular endothelial growth factor (VEGF), which is believed to be the central growth factor in angiogenesis. TGF-*β*1/ALK-5 stimulation elevated the m-RNA levels of VEGF in bovine aortic ECs, whereas BMP-9/ALK-1 stimulation led to decreased VEGF m-RNA levels. Proliferation and migration assays were in line with these observations [73]. 

 A remaining question is the interdependence between ALK-1 and ALK-5. Whereas Goumans et al. proposed that ALK-5 mediates a TGF-*β*-dependent recruitment of ALK-1 into the receptor complex and that ALK-5 kinase activity is essential for optimal ALK-1 activity [68]. Shao et al. observed opposite effects. They found some hints that ALK-1 acts independently of ALK-5 but that ALK-5 might actually be dependent of ALK-1 [73]. Hence, interdependence between ALK-1 and ALK-5 seems to be apparent, yet it has to be clarified which of the receptors is the leading force.

## 4. TGF-***β*** Receptor Expression in Leukemia

Since endothelial and hematopoietic cells have a common stem cell, the so-called hemangioblast, many immature hematopoietic cells share cell surface receptors with endothelial cells, such as receptors for hematopoieitc growth factors, for example, GM-CSF or erythropoietin [74, 75]. Our group investigated expression of ALK-1 and ALK-5 in various leukemic cells lines and samples from patients with acute myeloid leukemia (AML). We found that both receptors are expressed in most cases implying that both ALK-1 and ALK-5 are involved in autocrine or paracrine growth stimulation in AML (manuscript in preparation).

 In a recent study, an association between high ID-1 expression and poor prognosis in patients with AML has been described. ID-1 is the typical downstream mediator of ALK-1 signaling although enhancement of ID-1 expression by other tryrosine kinase receptors such as FLT3 cannot be excluded in a subgroup of patients [76]. However, data about dysregulated TGF-*β* signaling in hematologic malignancies are rare, since only few reports in lymphoid neoplasms or myeloid leukemia have been published [52, 53, 77, 78].

## 5. TGF-***β*** Signaling Pathway as a Therapeutic Target

Because of the enormous number of observed alterations in the TGF-*β* pathway in cancer patients, the development of therapeutic substances seems to be evident. 

**Figure 2:**
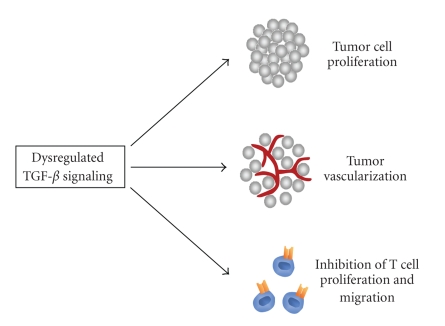
Dysregulation of the TGF-*β* pathway promotes tumor growth. An unbalanced TGF-*β* pathway can cause advanced tumorigenesis due to several cellular changes. On the one hand, the dysregulation has a direct effect on tumor cells leading to elevated tumor cell proliferation. Secondly, endothelial cells are affected which results in increased angiogenesis and therefore in tumor vascularization. Finally immune responses are attenuated due to inhibition of T cell proliferation and migration caused by dysregulated TGF-*β* signaling.

 In fact, there are different reasons why the inhibition of the TGF-*β* pathway might be a promising target for anticancer therapies. First, the direct effect on tumor cells has to be stressed. Secondly, as described above, the TGF-*β* pathway plays an important role in endothelial cell behavior and therefore in angiogenesis. Antiangiogenic therapies belong to the most promising therapeutic concepts which are currently under development. Thirdly, TGF-*β* is one of the most potent naturally immunosuppressors [79, 80]. Mice deficient for TGF-*β*1 develop a harmful syndrome with multifocal, mixed inflammatory cell response, and tissue necrosis, leading to organ failure and death [4]. Furthermore, suppression of TGF-*β* signaling in T cells by transduction with a truncated TGFBR2 resulted in severe autoimmune reactions [81]. The immune response of cancer patients is often suppressed, since many advanced tumors overexpress TGF-*β* resulting in inhibition of IL-2-dependant proliferation and differentiation of NK and T cells [82, 83]. In addition, TGF-*β* recruits different immune cells to the tumor microenvironment: monocytes and macrophages promote tumor invasion, angiogenesis and metastasis whereas mast cells secrete numerous tumor promoting factors [83]. Figure 2 summarizes the main tumor promoting effects of dysregulated TGF-*β* signaling. 

 Targeting the TGF-*β* pathway should therefore not only affect the tumor cells by itself; moreover a decreased tumor vascularization and strengthening the patient's immune responses should be achieved. Numerous in vitro and in vivo studies have been performed, accounting for these different strategies to inhibit tumor growth and to target various components within the TGF-*β* pathway including ligands, receptors and even downstream signals. Some of these studies passed the preclinical phase with success and phase I and II clinical studies have been started. Table 1 gives a short overview of preclinical and clinical studies using agents targeted at TGF-*β* family members. 

 Representing the central factor of the pathway, TGF-*β* is the preferred target structure in most cases. For example, Yang et al. developed transgenic mice expressing a TGF-*β* antagonist consisting of a soluble TGF-*β* type II receptor fused with the Fc domain of a human IgG1. The number of metastases was reduced both in a tail vein metastasis assay with melanoma cells and in crosses with a transgenic mouse model of metastatic breast cancer [101]. A neutralizing pan-TGF-*β* antibody prevented radiation-induced acceleration of metastatic cancer progression in a transgenic mouse model of metastatic breast cancer [102]. The pan-TGF-*β* antibody GC-1008 was tested in a phase I clinical study with 22 patients with renal cell carcinoma or malignant melanoma (NCT00356460). Treatment was well tolerated with mainly grade 1-2 toxicity including skin rash, fatigue, headache and gastrointestinal symptoms. 5 patients achieved stable disease or better and one patient with skin disease achieved a partial response with >75% reduction of target lesions [84]. 

 Several studies concentrated on the restoration of immune responses. In a prostate cancer xenograft model a reduction in tumor weight was observed after implantation of tumor-reactive CD8^+^ T cells which were TGF-*β* insensitive due to introduction of a dominant-negative TGF-*β* type II receptor vector [103]. Yamamoto et al. utilized direct hemoperfusion treatment with specific immunosuppressive substance adsorption columns for TGF-*β* ablation in rats bearing a TGF-*β*-producing hepatocellular carcinoma. TGF-*β* serum levels were decreased after hemoperfusion treatment leading to restored T lymphocyte response, decelerated tumor growth and longer survival times [104]. Fujita et al. observed similar results using plasmid DNA encoding the extracellular domain of the TGF-*β* type II receptor fused to the human IgG heavy chain; after plasmid injection in the proximity of established murine lymphomas an increased number of tumor antigen-specific CD4^+^ and CD8^+^ cells could be detected in tumor-draining lymph nodes [105]. 

 Another promising approach which has entered clinical phase I and II trials is to inhibit TGF-*β* function by means of antisense oligonucleotides (AS-ODNs). In a preclinical trial, local intracranial administration of TGF-*β*2 AS-ODNs was combined with systemic tumor vaccine in a rat glioma model. Only the combination of both substances led to a significantly prolonged survival [106]. Increased survival of glioma patients who had received whole-cell vaccines comprising autologous tumor cells genetically modified by a TGF-*β*2 antisense vector was observed in a phase I study [107]. A phase II trial with belagenpumatucel-L, a TGF-*β*2 antisense gene-modified allogeneic tumor vaccine, is ongoing in patients with advanced nonsmall cell lung cancer (NCT01058785) [87]. The antitumorigenic effect of antisense oligonucleotides was supported by phase II trials with the TGF-*β*2 inhibitor AP12009. In comparison to standard chemotherapy, treatment with AP12009 resulted in prolonged survival of patients with anaplastic astrocytoma [85]. Consistently, patients with high-grade glioma achieved a higher survival rate at 24 months and showed significantly more responders after 14 months when AP12009 treatment was compared to standard chemotherapy protocols [86]. 

 Therapeutic concepts against TGF-*β* receptors were almost exclusively targeted at ALK-5, in most cases using small molecule inhibitors such as SB431542 which showed similar results in several in vitro and in vivo studies. SB431542 caused inhibition of proliferation, TGF-*β*-mediated morphognic changes, and cellular motility of glioma cells in vitro. This effect was due to blocked phosphorylation of SMADs leading to reduced transcription of PAI-1 and VEGF which are key mediators in cell invasion and neoangiogenesis [95]. Javelaud and collegues analyzed the role of TGF-*β* in murine melanoma metastasis to bone. Both the therapy with SB431542 as well as tumors transduced with the inhibitory protein SMAD-7, showed significantly less osteolyses, longer survival and lower expression levels of osteolytic factors such as parathyroid hormone-related protein and interleukin-11 [96]. 

 Another ALK-5 small molecule inhibitor, SD208, led to decreased tumor growth and metastasis in a murine mamma carcinoma and pancreatic adenocarcinoma model. Furthermore, antiangiogenic effects could be observed in both studies with a reduced microvessel density and altered expression levels of angiogenesis-related factors like FLT-1, Neuropillin-2 and VEGF-C, respectively [97, 98]. In addition, treatment of CD34^+^ cells isolated from patients with myelodysplastic syndrome with SD208 led to in vitro enhancement of hematopoiesis [78]. Furthermore in a malignant mesothelioma mouse model, the ALK-5 inhibitor SM16 significantly decreased tumor growth which could be ascribed to a CD8^+^ antitumor response [99]. 

 The substance LY2109761 inhibits both TGF-*β* type I and type II receptors [88]. An orthotopic murine model of metastatic pancreatic cancer and a liver metastasis model proved the efficacy of LY2109761, since tumor growth and spontaneous metastases were reduced whereas the animals' survival was prolonged [89]. Similar effects were observed in an experimental colorectal cancer mouse model [90]. Gianelli's group performed several studies with LY2109761 in hepatocellular carcinoma. Tumor progression was delayed due to inhibition of vascular invasion as well as disturbance of cross-talk between hepatocellular carcinoma cells and stroma or endothelial cells. In a xenograft chick embryo model, LY210976 treatment caused even enhanced inhibition of tumor growth and reduced microvessel density compared to bevacizumab-treated animals. However, the strongest antitumoral effect was observed when combining both substances [91–93]. Myelo-monocytic leukemic cells cocultured with bone marrow derived mesenchymal stem cells were stimulated with TGF-*β*1 which led to inhibition of spontaneous and cytarabine-induced apoptosis. This prosurvival signaling was neutralized with LY2109761 [94].

 Although no in vitro data about specific ALK-1 inhibitors have been published so far, a clinical phase I study testing a human antiALK-1 antibody in patients with advanced solid tumors is ongoing (NCT00557856). 

 Since its expression is restricted to endothelial cells with higher expression levels in tumor-associated endothelium compared to normal tissue, the accessory receptor endoglin may represent a promising target for anticancer therapy [108]. Antitumorigenic and antiangiogenic effects could be observed in several in vivo tumor models using antiendoglin antibodies [109–112]. For example, Uneda et al. used multiple metastasis models with murine mamma carcinoma and colon adenocarcinoma cells to test the effect of several antiendoglin antibodies targeted at different endoglin epitopes. Under treatment, metastases were suppressed and microvessel density was effectively reduced as measured by Matrigel plug assay [109, 113]: a phase I clinical trial with the human/murine chimeric antiendoglin monoclonal antibody TRC105 in 19 patients with solid cancer. Treatment was well tolerated with mainly grade 1-2 toxicity including fatigue, anemia, proteinuria and diarrhea. One patient with hormone refractory prostate cancer obtained a complete PSA response and 3 patients had prolonged stable disease (NCT00582985) [100].

## 6. Outlook

The results of numerous in vitro studies with cell lines, in vivo mouse models and clinical trials show that the TGF-*β* pathway plays an important role in cancer progression and represents a promising target for anticancer therapy. Targeting TGF-*β* isoforms, TGF-*β* receptors as well as downstream signaling proteins yielded satisfactory results, since a reduction in tumor load was observed in most cases. 

 Manipulating TGF-*β* signaling implies the great advantage of affecting at least three important structures in tumor progression: in addition to the direct antitumor effect, endothelial and immune cells will be targeted. Although restoration of the immune system is a desirable achievement in cancer therapy, the complete inhibition of TGF-*β*1 might have fatal consequences. For example, TGF-*β*1 deficient mice develop a lethal syndrome accompanied by a multifocal, mixed inflammatory cell response and tissue necrosis, leading to organ failure [4]. Furthermore, abrogation of TGF-*β* signaling in T cells by introduction of a truncated TGFBR2 results in severe autoimmune reactions [81].

 In addition, TGF-*β*1 plays an important role in fibroblast biology, since it is a relevant growth factor for extracellular matrix formation in fibroblasts due to its stimulation of collagen, fibronectin and proteoglycan synthesis [114]. Due to TGF-*β* signaling, fibroblasts suppress the activation of tumor-promoting paracrine signaling which would target epithelial cells and could lead to epithelial to mesenchymal transition [115]. Bhowmick et al. inactivated the TGFBR2 gene in mouse fibroblasts which resulted in intraepithelial neoplasia in prostate and invasive squamous cell carcinoma of the forestomach [115]. Coimplantation of TGFBR2-deficient mammary fibroblasts with mammary carcinoma cells promoted tumor growth and invasion as compared to wild-type fibroblasts [116]. 

 These examples reveal the great difficulty in targeting the TGF-*β* pathway. Perhaps targeting the type I receptor ALK-1 or the accessory receptor endoglin might represent a solution to this discrepancy since expression of both receptors seems to be restricted to endothelial cells which could limit side effects. Therefore, results of phase I studies are awaited where patients with advanced solid tumors will be treated with an ALK-1 or an endoglin antibody (NCT00557856 and NCT00582985, resp.). These studies might resolve this question. 

 Integrating all results underlines the complexity in TGF-*β* signaling in endothelial cells. In some extent, this may be due to different experimental settings since TGF-*β* superfamily members have often been overexpressed or downregulated in in vitro models using plasmid vectors. Furthermore, although discussed as important ALK-1 ligand in the regulation of angiogenesis, no physiological data about BMP-9 or BMP-10 expression in endothelial or tumor cells exist.

## 7. Conclusion

We conclude that the TGF-*β* pathway might be a promising therapeutic target in anticancer therapy due to its involvement in several mechanisms including endothelial and immune cell biology that are most important for tumor progression. On the other hand, since the TGF-*β* pathway affects a broad range of cellular behavior, it is an ambitious approach to restore the delicate balance of physiological signaling. Therefore manipulation of the pathway bears the risk of adverse effects and of therapeutic success. Comprehensive investigations that comprise the interactions between tumor cells, fibroblasts, endothelial and immune cells are indispensable.

## Figures and Tables

**Table 1:** Overview of preclinical and clinical studies using agents targeted at TGF-*β* family members.

Class of substance	Target	Drug	Study
Human anti-TGF-*β* mAb	TGF-*β*	GC1008	Phase I study on renal cell carcinoma and malignant melanoma (NCT00356460 and NCT00899444) [84]
TGF-*β*2 antisense compound	TGF-*β*2	AP12009	Phase I study on pancreatic and colorectal neoplasms and melanoma (NCT00844064)
			Phase II study on glioblastoma and anaplastic astrocytoma (NCT00431561) [85, 86]
			Phase III study on anaplastic astrocytoma (NCT00761280)
		Belagenpumatucel-L	Phase II study on advanced nonsmall lung cancer (NCT01058785) [87]
TGF-*β* type I and type II receptor small molecule inhibitor	TGF-*β* type I and type II receptors	LY2109761	Preclinical studies [88–94]
Human anti-ALK-1 mAb	ALK-1	PF-03446962	Phase I on advanced solid tumors (NCT00557856)
ALK-5 small molecule inhibitor	ALK-5	SB431542	Preclinical studies [95, 96]
		SD208	Preclinical studies [97, 98]
		SM16	Preclinical studies [78, 99]
Chimeric antiEndoglin antibody	Endoglin	TRC105	Phase I on advanced or metastatic solid cancer (NCT00582985) [100]
